# Effects of cranberry powder on the diversity of microbial communities and quality characteristics of fermented sausage

**DOI:** 10.3389/fnut.2023.1123627

**Published:** 2023-04-11

**Authors:** Le Yang, Xinlei Yan, Ting Liu, Letian Kang, Yufei Sun, Xingyu Gao, Xin Zhao, Yan Duan

**Affiliations:** ^1^College of Food Science and Engineering, Inner Mongolia Agricultural University, Hohhot, China; ^2^Integrative Research Base of Beef and Lamb Processing Technology, Hohhot, China

**Keywords:** fermented sausage, cranberry powder, high-throughput sequencing, bacterial diversity, volatile flavor compounds

## Abstract

Fermented sausage is popular with many consumers because of its distinctive flavor, but the safety of it has attracted widespread attention. At present, nitrite is widely used in fermented meat products because of its ideal color and bacteriostatic effect, but nitrite can be transformed into nitrosamines, which cause strong carcinogenic effects. Therefore, it is urgent to actively explore safe and efficient nitrite substitutes. In this study, cranberry powder was selected as a natural substitute for nitrite during the production of fermented sausage due to its unique antioxidant and bacteriostatic properties. The results showed that adding an appropriate amount of cranberry powder (5 g/kg) promoted a better color of the fermented sausage and promoted the accumulation of aromatic compounds. Furthermore, *Pediococcus* and *Staphylococcus* became the dominant species, accounting for more than 90% in all samples. According to the Pearson correlation analysis, *Staphylococcus* and *Pediococcus* had positive effects on the quality characteristics of fermented sausage products. This study provided the latest information on the application of cranberry powder as a natural substitute for nitrite in the process of manufacturing fermented sausage, and it also introduced an advanced solution to improve the quality characteristics and safety of fermented sausage products during processing.

## 1. Introduction

Fermented sausage is a traditional fermented food that is widely produced because of its unique flavor and nutrition. However, it is difficult to guarantee the quality stability and safety of traditional sausage that is naturally fermented (1, 2). To realize the industrialized production of fermented sausage, meet consumers’ desire of food safety and health, and ensure the flavor quality of fermented sausage, artificial inoculation fermentation has attracted much attention. Inoculating starters can shorten the ripening time, improve the safety, stability, and shelf life and enhance the flavor of fermented sausage (3–6). In addition, the structure and composition of bacteria in fermented sausage play a decisive role in product quality. Zhang et al. revealed that the use of salt substitutes during fermentation and ripening changed the composition of the bacterial community and reduced the diversity of the bacterial community in low-sodium fermented sausage based on the analysis of bacterial community composition and physicochemical and sensory characteristics (7). Thus, it is clear that different fermentation methods and fermentation agents directly affect the bacterial community and structure of fermented sausage (8, 9).

Nitrates and nitrite additives are used in the processing of fermented meat products, which can inhibit the growth and reproduction of spoilage microorganisms, cause the effect of antioxidation that produces an ideal color and play an important role in the production of flavor (10). However, the degradation of fermented sausage protein promotes the generation of some biogenic amines, and excessive biogenic amines react with residual nitrite in meat products to produce carcinogenic nitrosamines (11–14). Consequently, research on natural substances to substitute nitrites is being performed more than before, and adding some natural ingredients to reduce the residual amount of nitrite is a direct way to improve the safety of meat (15, 16). Some scholars have found that many natural substances have a certain effect of removing nitrite, and these substances include ascorbic acid (VC) and various natural plant extracts, because these plants are rich in reducing substances such as VC, flavonoids, phenols, etc. (17, 18).

Cranberry (*Vaccinium macrocarpon*) is a natural food and medicinal plant that has attracted much attention in recent years because of its unique health and medicinal effects. It is currently widely used in powder and extract forms in a variety of food and nutritional supplements (19, 20). Some studies have shown that high concentrations of cranberry polyphenols are associated with antibacterial, antiviral, anti-inflammatory and antioxidant properties (21–24). Caldas et al. emphasized that cranberry polyphenols were potent antioxidants that can eliminate free radicals and reduce cellular damage. The mechanism was that cranberry polyphenols could neutralize reactive oxygen species directly and were able to interfere with some cell signaling pathways indirectly (25). However, there has been no relevant study on cranberry powder used as a natural substitute for nitrite in fermented sausage until now. In this work, we aimed to investigate the effects of adding different contents of cranberry powder to fermented sausage, as well as the relationship between volatile flavor components and other quality characteristics of fermented sausage with its dominant microbiota.

## 2. Materials and methods

### 2.1. Preparation of the fermented sausage

All meat and ingredients were purchased from a supermarket (Saihan District, Hohhot, China). The ingredients in the traditional formulation (glucose 0.5%, salt 2.5%, sucrose 0.5%, and black pepper 0.5%) were added to a mixture of 80% chopped sheep hind leg and 20% sheep tail fat. Fermented sausage was inoculated with a recommended commercial starter culture composed of *Pediococcus pentosaceus* and *Staphylococcus xylosus* (F-1 BactofermTM, Chr-Hansen, Denmark) for traditionally fermented meat products, with an amount of 25 g/200 kg. The production of the fermented sausage was divided into four experimental groups according to the experimental design. Each experimental group was stored in parallel for three batches in lab and the average weight of the final produced sausages is about 35 g. The control group (CK) was given 100 mg/kg sodium nitrites and no cranberry powder, while the other three experimental groups were added 3 g/kg (M3), 5 g/kg (M5), 7 g/kg (M7) cranberry powder and 50 mg/kg sodium nitrites, respectively. Then the mixture was stuffed into natural casings with a diameter of 10 cm. Fermentation was performed at 25°C and 95% relative humidity (RH) for the first 4 days. For ripening process, the temperature was set at 16°C and 90–70% RH for 8 days. After maturation, the samples were independently packed in a vacuum and stored at 4°C for 0 days, 1 day, 4 days, 8 days and sausage sampling (50 g) was performed in triplicate immediately stored at −80°C for subsequent analysis.

### 2.2. Physicochemical analysis

Water activity (aw) was measured using an HD-3A water activity meter (Wuxi Huake Instrument & Meter Co. Ltd.) at 25°C. The sample was chopped up and placed in the water activity apparatus (capacity: 20 mL).

The shredded sample (2 g) was put in 18 mL of normal saline (0.85% NaCl, v/v), stood for 15–20 min and then filtered and set aside. The pH value of the homogenized sample was determined using a PB-10 pH meter (Sartorius, AG, Germany). The pH meter was calibrated with standard solution (pH 4.0 and 7.0).

Color parameters of sausage were carried out with TCP2 automatic color meter (Beijing Xin’ao Yike Photoelectricity Technology Co., Ltd.) and expressed as L*(lightness-darkness), a*(redness), and b* (yellowness). The sausage products were cut into slices about 0.5 cm thick and each sample was repeated 3 times individually. The color meter was preheated for 15 min and calibrated with standard plates *Y* = 87.3, *X* = 0.3158 and *Y* = 0.3233 before detection.

### 2.3. Texture profile analysis

The texture profile was analyzed by a texture analyzer (QTS texture instrument: FTC Corporation, USA). The determination parameters were set as follows: pre-test rate, 2 mm/s; pilot test rate, 2 mm/s; post-test rate, 2 mm/s; test interval, 5 s; compression ratio, 50%; trigger force, 5 g; trigger type, automatic. Samples were cut into cubes (length, width and height 1 cm) to fit on the platform. The hardness (N), springiness, adhesiveness (g·sec), gumminess, chewiness (N) and resilience were measured by two cycles at room temperature by using a “P-100” probe (26). The samples in each group were determined five times in parallel.

### 2.4. Microbiological analysis

Microbiological analysis was according to the method described by Yu et al. (27) with some modifications. Samples were randomly taken from three places in each group and 5 g was taken from each place. After mixing chopped meat samples, 10 g was taken from them for microbiological experiment. The aerobic bacteria were identified on a plate count agar (PCA, Nanjing Jianceng Biotechnology Co., Ltd., Nanjing, China, dilution solution: distilled water) after incubation at 37°C for 48 h. The data were expressed as Log CFU/g.

### 2.5. Volatile compounds analysis

The HS-SPME and GC–MS system (ISQ GC–MS instrument, Thermo Fisher Scientific, USA) were employed for the extraction of volatile compounds from the fermented sausages. The extraction was referred to the method proposed by Luo. (28).

### 2.6. Illumina MiSeq sequencing

#### 2.6.1. DNA extractions

DNA from different samples which were frozen with liquid nitrogen and ground into powder was extracted using the E.Z.N.A. ® Soil DNA Kit (Omega, Inc., USA) in accordance with the manufacturer’s instructions. Nuclear-free water was used as the blank. The total DNA was eluted in 50 μl of Elution buffer and stored at −80°C before the PCR amplification.

#### 2.6.2. PCR amplification

The V3–V4 regions (469 bp) of the 16S rRNA gene were amplified using the primers 341F (5′-CCTACGGGNGGCWGCAG-3′) and 805R (5′-GACTACHVGGGTATCTAATCC-3′). PCR amplification was performed in a total volume of 25 μl with 12.5 μl of 2 × Taq Master Mix, 2.5 μl of each primer, 25 ng of template DNA. The PCR conditions to amplify the prokaryotic 16S fragments consisted of an initial denaturation at 98°C for 30 s; 35 cycles of denaturation at 98°C for 10 s, annealing at 54°C/52°C for 30 s, and extension at 72°C for 45 s; and then final extension at 72°C for 10 min.

#### 2.6.3. 16S rDNA sequencing

Throughout the DNA extraction process, ultrapure water, instead of a sample solution, was used to exclude the possibility of false-positive PCR results as a negative control. The PCR products were purified by AMPure XT beads (Beckman Coulter Genomics, Danvers, MA, USA) and quantified by Qubit (Invitrogen, USA). The amplicon pools were prepared for sequencing and the size and quantity of the amplicon library were assessed on Agilent 2,100 Bioanalyzer (Agilent, USA) and with the Library Quantification Kit for Illumina (Kapa Biosciences, Woburn, MA, USA), respectively. The libraries were sequenced on the NovaSeq PE250 platform.

### 2.7. Bioinformatics analysis

The samples were sequenced on an Illumina NovaSeq PE250 platform according to the manufacturer’s recommendations, provided by LC-Bio. Paired-end reads were assigned to samples based on their unique barcode and truncated by cutting off the barcode and primer sequence. Paired-end reads were merged using FLASH. Quality filtering on the raw reads was performed under specific filtering conditions to obtain the high-quality clean tags according to the fqtrim (v0.94). Chimeric sequences were filtered using Vsearch software (v2.3.4). After dereplication using DADA2, we obtained feature table and feature sequence. Alpha diversity and beta diversity were calculated by normalized to the same sequences randomly. Then according to SILVA (release 138) classifier, feature abundance was normalized using relative abundance of each sample. Sequences with ≥97% similarity were assigned to the same operational taxonomic units (OTUs) by Vsearch (v2.3.4) (29). Alpha diversity is applied in analyzing complexity of species diversity for a sample through 5 indices, including Chao1, Observed species, Goods coverage, Shannon, Simpson, and all those indices in our samples were calculated with QIIME2. Beta diversity was calculated by QIIME2, the graphs were drawn by R package. Blast was used for sequence alignment, and the feature sequences were annotated with SILVA database for each representative sequence. Other diagrams were implemented using the R package (v3.5.2).

### 2.8. Statistical analysis

Data were presented as mean ± standard deviation (SD.). All samples were carried out in triplicate (three independent productions). The statistical significance (*p* < 0.05) was analyzed by one-way ANOVA (Analysis of Variance) and Tukey’s posthoc test. SPSS 22.0 (SPSS Inc., Chicago, IL, USA) was used for all statistical analyses. Correlation Network was performed using the OmicStudio tools at https://www.omicstudio.cn/tool.

## 3. Results

### 3.1. Physicochemical properties of fermented sausage

The analysis on the physicochemical properties of the fermented sausages was shown in Table 1. The results showed that the values of a_w_, pH, L*, a* and b* in the four groups exhibited a decreasing trend with increasing fermentation time. At the initial stage of producing fermented sausage (Day 0), the values of a_w_ in the experimental groups (M3, M5, and M7) were significantly higher than those in the control group (CK; *p* < 0.05). The pH value of samples in the M5 group was significantly higher than that in the other cranberry powder groups at the finished product stage (8 days). The values of the L*, a*, and b* parameters obtained in our study showed that the a* value of M5 was significantly higher than that of the other groups in the finished product period (8 days; *p* < 0.05). In addition, the b* value of fermented sausage samples in other experimental groups except for CK at 8 days was significantly lower than that in other periods (*p* < 0.05).

**Table 1:** Physicochemical properties of fermented sausages.

Indexes	Group	Fermentation times (days)			
		0d	1d	4d	8d
a_w_	M3	0.9 ± 0.002Aa	0.9 ± 0.003Aa	0.8 ± 0.005Aa	0.7 ± 0.072Ba
	M5	0.9 ± 0.001Aab	0.9 ± 0.002Bb	0.8 ± 0.004Cab	0.7 ± 0.007 Da
	M7	0.9 ± 0.001Aa	0.9 ± 0.001Ba	0.8 ± 0.009Cb	0.7 ± 0.006 Da
	CK	0.9 ± 0.001Ab	0.9 ± 0.001Bb	0.8 ± 0.002Cc	0.7 ± 0.001 Da
pH	M3	6.2 ± 0.02Aa	4.5 ± 0.02Bb	4.5 ± 0.02Ba	4.2 ± 0.02Cc
	M5	6.0 ± 0.02Ac	4.5 ± 0.02Bb	4.4 ± 0.02Cab	4.3 ± 0.02 Da
	M7	6.0 ± 0.02Ab	4.4 ± 0.02Bb	4.4 ± 0.01Bb	4.3 ± 0.01Cb
	CK	5.9 ± 0.01Ad	4.5 ± 0.03Ba	4.4 ± 0.02Cab	4.3 ± 0.02 Da
L^*^	M3	42.9 ± 0.21Ca	44.3 ± 0.39Ba	45.0 ± 0.21Aa	35.7 ± 0.24Dc
	M5	37.4 ± 0.60Cc	40.6 ± 1.14Bb	44.5 ± 1.45Aa	39.0 ± 0.22BCa
	M7	37.5 ± 0.48Cc	41.5 ± 0.47Bb	44.1 ± 0.39Aa	36.8 ± 0.37Cb
	CK	41.8 ± 0.60Cb	43.7 ± 0.50Ba	45.1 ± 0.38Aa	38.7 ± 0.27 Da
a*	M3	22.1 ± 0.40Ac	19.8 ± 0.20Bb	18.4 ± 0.16Ca	15.5 ± 0.47Db
	M5	25.4 ± 0.28Aa	22.9 ± 0.12Ba	17.8 ± 0.27Ca	16.4 ± 0.15 Da
	M7	24.4 ± 0.46Ab	23.2 ± 0.61Ba	18.3 ± 0.84Ca	15.3 ± 0.20Db
	CK	16.6 ± 0.68ABd	17.5 ± 0.48Ac	16.0 ± 0.28BCb	15.2 ± 0.70Cb
b*	M3	7.7 ± 0.70Aa	6.9 ± 0.58Aa	7.5 ± 0.24Aa	4.6 ± 0.25Ba
	M5	6.9 ± 0.22Ab	6.8 ± 0.12Aa	6.5 ± 0.30Ab	4.4 ± 0.19Ba
	M7	6.29 ± 0.359Ab	6.05 ± 0.159Ab	5.66 ± 0.21Ac	4.73 ± 0.695Ba
	CK	7.94 ± 0.11Aa	7.32 ± 0.33Ba	7.08 ± 0.523Bab	4.75 ± 0.181Ca

Mean ± SD (*n* = 3). Different capital letters represent significant differences in different periods of the same group (*p* < 0.05), different lowercase letters represent significant differences between groups at the same time (*p* < 0.05), while the same letter indicated no significant difference (*p* > 0.05).

### 3.2. Texture profile analysis of fermented sausage

In this study, the hardness (N), springiness, adhesiveness (g·sec), gumminess, chewiness (N) and resilience indices were selected to determine the texture of fermented sausage. During the period of fermenting the sausage (8 days), the hardness value of fermented sausage samples in the experimental groups with cranberry powder was significantly higher than that in the control group without cranberry powder (*p* < 0.05; Table 2.). In addition, the hardness value of M5 with 5 g cranberry powder was the highest, and the hardness value of M3 with 3 g cranberry powder was the lowest among the experimental groups. In addition, the springiness, gumminess and chewiness of the samples in the M5 group were significantly higher than those in the other groups (*p* < 0.05), and there were no significant differences in the resilience indices among the four groups (*p* > 0.05).

**Table 2:** TPA of fermented sausage at 8 days.

Group	Characteristic indexes					
	Hardness (N)	Springiness	Adhesiveness (g·sec)	Gumminess	Chewiness (N)	Resilience
M3	2,970 ± 335AB	0.6 ± 0.01B	0.5 ± 0.06A	1,533 ± 380B	882 ± 207B	0.1 ± 0.02A
M5	3,408 ± 499A	0.8 ± 0.14A	0.5 ± 0.04A	2,553 ± 487A	18,978 ± 946A	0.2 ± 0.03A
M7	3,369 ± 441A	0.5 ± 0.17B	0.5 ± 0.06A	2007 ± 312B	1,103 ± 239B	0.1 ± 0.03A
CK	2,728 ± 312B	0.6 ± 0.07B	0.5 ± 0.02A	1,590 ± 382B	823 ± 259B	0.1 ± 0.01A

Mean ± SD (*n* = 3); Different letters represent significant differences between groups (*P* < 0.05), while the same letter indicated no significant difference (*P* > 0.05).

### 3.3. Microbiological analysis

The microbial counts of the fermented sausage during fermentation were presented in Table 3. The counts of aerobic bacteria in each group showed a fluctuating trend of increasing first, then decreasing and then increasing with time. The number of aerobic bacteria in each group at 8 days was significantly higher than that in the other periods (*p* < 0.05), and the number of aerobic bacteria in the experimental group with 5 g cranberry powder was significantly higher than that in the other experimental groups (M3, M7; *p* < 0.05).

**Table 3:** Changes of total viable bacteria number during fermentation.

Group	Fermentation times (days)			
	0d	1d	4d	8d
M3	6.38 ± 0.03 Da	8.64 ± 0.03Ba	8.17 ± 0.035Cc	8.73 ± 0.051Ab
M5	6.52 ± 0.15 Da	8.47 ± 0.045Bb	8.22 ± 0.04Cc	8.82 ± 0.02Aa
M7	6.52 ± 0.31Ca	8.6 ± 0.031Ba	8.53 ± 0.04Ba	8.67 ± 0.02Ac
CK	6.53 ± 0.086 Da	8.61 ± 0.1Ba	8.46 ± 0.01Cb	8.86 ± 0.02Aa

Mean ± SD (*n* = 3); Different capital letters represent significant differences in different periods of the same group (*P* < 0.05), Different lowercase letters represent significant differences between groups at the same time (*p* < 0.05), while the same letter indicated no significant difference (*P* > 0.05).

### 3.4. Volatile compound analysis

The relative concentrations of the volatile flavor compounds in M3-, M5-, M7-, and CK-fermented sausage following GC–MS are shown in Supplementary Table 1. A total of 76 volatile compounds were detected during fermentation, comprising 15 alcohols, 8 aldehydes, 9 acids, 5 esters, 2 phenols, 3 ketones, 27 terpenes and 7 others (Supplementary Table 1). During the four fermentation periods, there were some fluctuations in the volatile flavor composition. Except for M3, the species of flavor compounds in the other groups decreased first and then increased with the change in fermentation time. At the product period (8 days), a total of 69 flavor compounds were identified and quantified in the M5 samples. In addition, 65, 62, and 57 volatile flavors were detected in the M3, M5, and CK samples, respectively. Compared to the cranberry powder groups, the control group (CK) had relatively fewer volatile flavor substances (57). The maximum amounts of alcohols, acids, esters and ketones were observed on Day 8 for each sample. At the same time, the samples in the M5 group had higher contents of various flavor substances than those in the other groups at 8 days.

As shown in Supplementary Table 1, various volatile compounds were detected in each group with different changes over time. Considering the total occurrence of the compounds, terpenes were the largest class of volatile flavor compounds determined from all samples. The contents of γ-terpinene and caryophyllene were significantly higher in the cranberry powder group samples than in the CK samples at 4 days (*p* < 0.05). Simultaneously, among the volatile compounds, ketones were the second-largest components found in the samples. Acetoin was detected in all samples, an upward trend was observed from the end of fermentation (4 days) to the finished product stage (8 days), and the content of acetoin in the M5 group was higher than that in the other groups. Furthermore, 2,3-butanediol and acetic acid content dominated the volatile components in amounts produced for samples at 8 days in other classes of substances. The flavor compound 2,3-butanediol was not detected at the first two time points in the four groups, and the content in the experimental group (M3, M5, and M7) was significantly higher than that in the control group (CK; *p* < 0.05) at 4 days. Otherwise, the aldehydes identified from samples included saturated aldehydes such as pentanal, hexanal, heptanal, nonanal and decanal, unsaturated aldehydes such as (E)-2-decenal, and aromatic aldehydes such as benzaldehyde and benzeneacetaldehyde.

### 3.5. Characteristics of the sequencing data comparison and community comparison

A total of 3,029,968 high-quality and valid sequences were recovered from 48 samples, with an average sequence length of 469 bp. The sequences of the bacterial 16S rRNA gene ranged from 50,999 to 72,618. The alpha diversity of the bacterial community structure in samples that involved the observed OTUs, Chao1, Simpson and Shannon indices. The coverage index in all samples was over 0.99, indicating that a sufficient bacterial diversity in the samples was detected.

The results (Table 4) showed that OTUs, Chao1 and Shannon indices in M3, M7, and CK decreased gradually in the processing process, and the samples of four groups at 0 days were significantly higher than those in each period (*p* < 0.05), indicating that the bacterial community richness and diversity of fermented sausage gradually decreased. The bacterial richness (OTUs =428.67 ± 48.99, Chao1 = 435.41 ± 53.53) of M5 on Day zero was significantly higher than that in other periods, and the bacterial diversity of the test group first decreased and then increased with increasing processing time. In addition, the bacterial community richness (OTU = 469.00 ± 70.68, Chao1 = 472.89 ± 71.69) and diversity (Shannon =3.16 ± 0.49, Simpson =0.64 ± 0.07) of M7 were higher than those of the other groups at the initial stage of fermented sausage processing (0 days). At the end of fermentation (4 days), the Shannon index of M3 was higher than that of the other groups, indicating that the bacterial colony diversity was higher than that of the other groups. After fermentation (8 days), the Shannon index and Simpson index of M5 were significantly higher than those of the other groups (*p* < 0.05).

**Table 4:** Different estimator indexes for alpha diversity in samples.

Times	Group ID	Observed OTUs	Shannon	Simpson	Chao1
0d	M3	437 ± 17Aab	2.80 ± 0.05Aab	0.62 ± 0.02Aa	441 ± 17Aab
	M5	429 ± 49Aab	2.53 ± 0.22Aab	0.56 ± 0.06ABa	435 ± 54Aab
	M7	469 ± 71Aa	3.16 ± 0.49Aa	0.64 ± 0.07Aa	473 ± 72Aa
	CK	338 ± 67Ab	2.28 ± 0.28Ab	0.58 ± 0.08Aa	345 ± 66Ab
1d	M3	101 ± 28Ba	1.21 ± 0.51Ba	0.38 ± 0.22ABa	101 ± 29Ba
	M5	108 ± 25Ba	1.30 ± 0.14Ca	0.39 ± 0.08Ba	109 ± 27Ba
	M7	99 ± 37Ba	1.34 ± 0.18Ba	0.40 ± 0.10Ba	100 ± 38Ba
	CK	98 ± 35Ba	1.46 ± 0.13Ba	0.53 ± 0.04Aa	100 ± 36Ba
4d	M3	97 ± 20Ba	1.68 ± 0.11Ba	0.57 ± 0.02ABa	99 ± 18Ba
	M5	104 ± 27Ba	1.43 ± 0.25Cab	0.50 ± 0.14ABa	106 ± 27Ba
	M7	143 ± 73Ba	1.49 ± 0.04Bab	0.48 ± 0.08ABa	147 ± 80Ba
	CK	94 ± 13Ba	1.35 ± 0.16Bb	0.48 ± 0.11Aa	95 ± 13Ba
8d	M3	148 ± 53Ba	1.25 ± 0.26Bb	0.32 ± 0.13Bb	150 ± 54Ba
	M5	96 ± 5Ba	1.90 ± 0.30Ba	0.64 ± 0.07Aa	96 ± 4Ba
	M7	136 ± 35Ba	1.48 ± 0.29Bab	0.47 ± 0.18ABab	138 ± 34Ba
	CK	157 ± 56Ba	1.23 ± 0.31Bb	0.37 ± 0.18Aab	161 ± 57Ba

Mean ± SD (*n* = 3); Different capital letters represent significant differences in different periods of the same group (*P* < 0.05), Different lowercase letters represent significant differences between groups at the same time (*p* < 0.05), while the same letter indicated no significant difference(*P* > 0.05).

**Figure 1:**
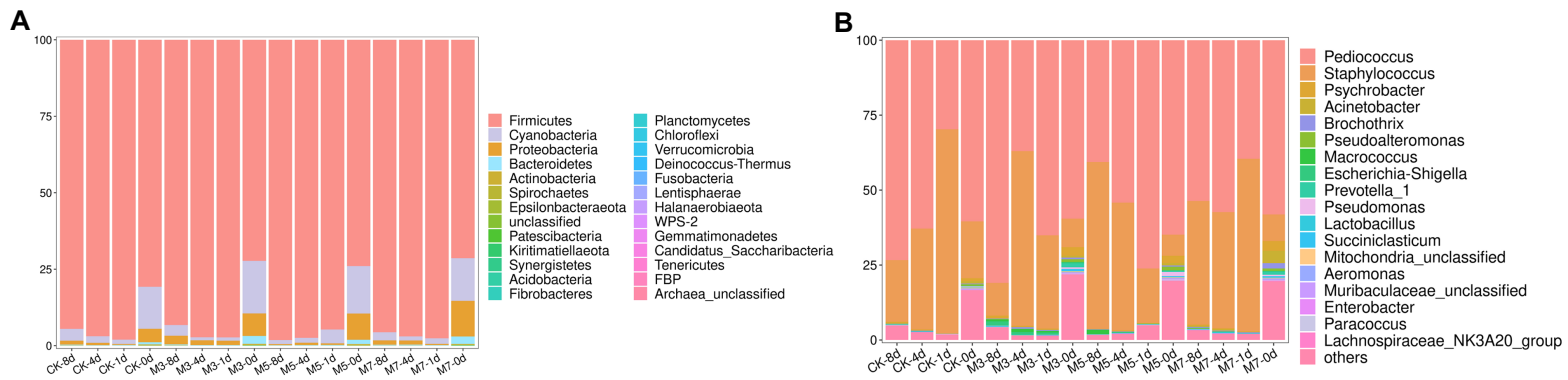
Relative abundance of bacteria community proportions based on 16S rDNA sequencing at phylum **(A)** and genus **(B)** level.

The bacterial communities of the M3, M5, M7, and CK samples were analyzed at the phylum (Figure 1A) and genus levels (Figure 1B). In all samples, there were mainly 5 identified phyla, namely, *Firmicutes*, *Cyanobacteria*, *Proteobacteria*, *Bacteroidetes*, and *Actinobacteria*, and 31 identified genera were found during fermentation. Moreover, *Firmicutes* was the dominant phylum, which ranged from 71.43 to 98.16%. The abundance of *Firmicutes* in the M5 group increased with increasing fermentation time (1–8 days), and the other groups showed a downward trend. However, there was no obvious difference in the composition of the bacterial community between the treatments at the phylum level.

The composition analysis of the bacterial community in the fermented sausages at the genus level is shown in Figure 1B. The genera *Pediococcus*, *Staphylococcus*, *Psychrobacter*, and *Acinetobacter* were the most representative bacteria. Moreover, *Pediococcus* and *Staphylococcus* in *Firmicutes* were abundant genera, accounting for more than 90% in all samples. However, the changing trend of bacterial community composition in each group was different. Compared with the M5, M7, and CK groups, *Pediococcus* composed the vast majority of the bacterial community of samples in the M3 group (*Pediococcus*: 80.90%; *Staphylococcus*: 10.95%) at the end of processing (8 days). At the same time, the proportion of *Staphylococcus* in the M5 group (*Staphylococcus*: 55.64%; *Pediococcus*: 40.67%) was higher than that in the other groups at 8 days. And according to Supplementary Figure 1, potential spoilage organisms in the genera *Psychrobacter*, *Acinetobacter*, *Brochothrix*, and *Pseudoalteromonas* were present at various abundances at all fermentation stages in the four groups. The total proportion of these spoilage microorganisms in each group decreased with increasing fermentation time. In the cranberry powder groups, the proportion of spoilage microorganisms in the M5 group was lower than that in the other two groups (M3, M7) at 8 days (Supplementary Figure 1E). In addition, the spoilage microorganisms in the M5 group were lower than those in the control group (CK) at the finished product stage (8 days).

**Figure 2:**
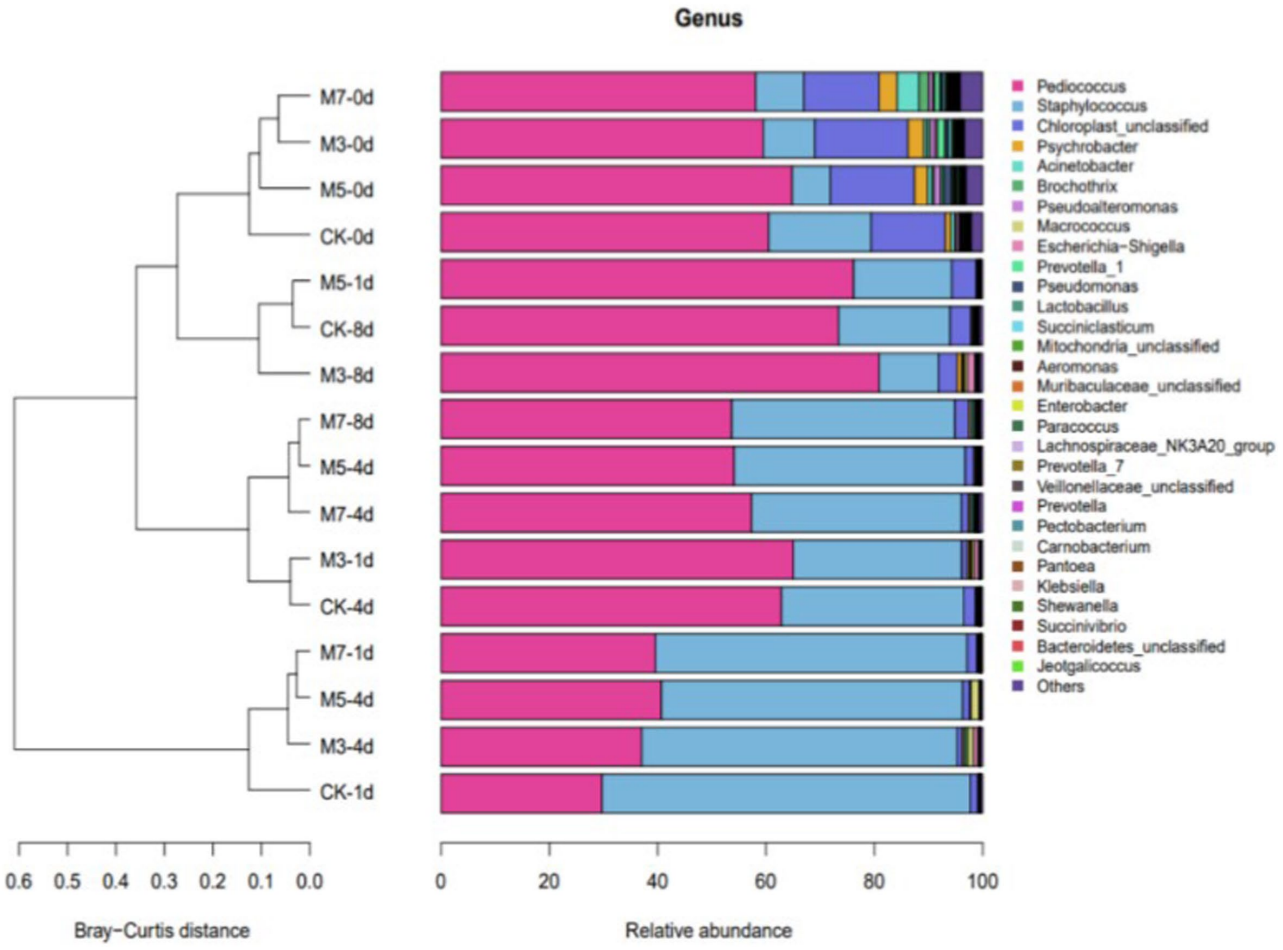
The Bray–Curtis distance method was based on operational taxonomic units (OTUs) of the bacterial community composition at the genus level in samples.

To reveal the effects of the amount of cranberry powder addition on the bacterial diversity of fermented sausage, the beta diversity among 48 samples was clustered by the Bray–Curtis distance method (Figure 2). In sample classification, the more similar the bacterial colony composition of samples was, the smaller the Bray–Curtis distance. For the bacterial diversity composition, the results showed that all samples were divided into two parts, and one part consisting of initial sausage samples was separated from the other samples. Specifically, the samples of M3 (0 days), M5 (0 days), CK (1 day), and M7 (1 day) were clustered together, and the remaining samples were divided into groups. According to the values in Figure 2, the composition of bacteria in all groups was similar only at Day 0 of fermentation.

**Figure 3:**
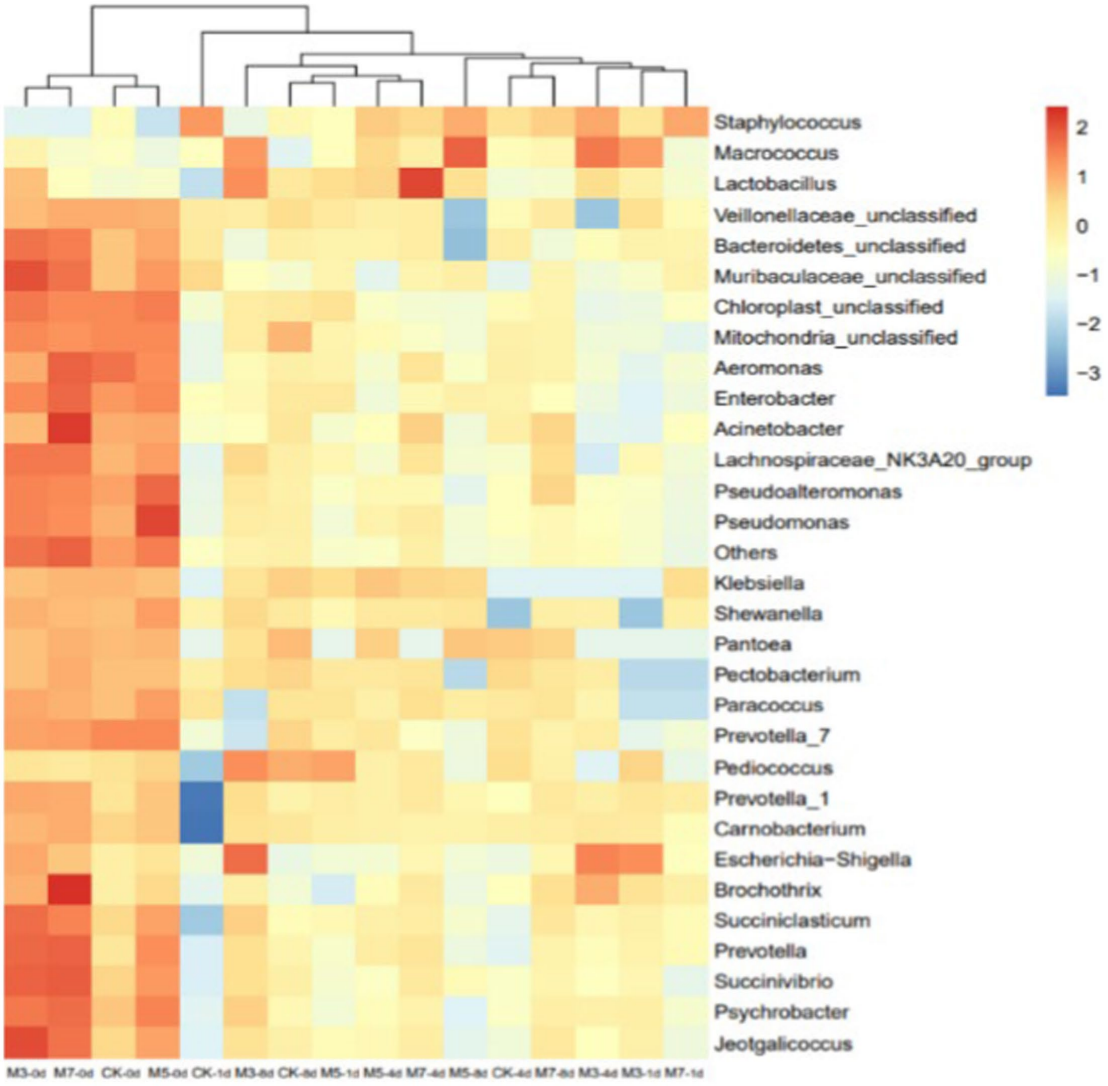
Bacterial community heatmap analysis and sample similarity tree at genus-level phylotype. In the heat map, red and blue represented high and low relative abundances, respectively.

To compare the diversity of bacterial composition in the four groups, bacterial diversity heatmap analysis was performed (Figure 3). Each column in the heatmap represents a different sample that was studied. The bacterial microbiota species diversity of the four groups at 0 days was similar and significantly higher than that at other stages, as shown in Figure 3. Furthermore, the bacterial distribution of the CK, M3, M5 and M7 samples at 1 day was not abundant, showing that the main bacterial microflora were *Staphylococcus* and *Pediococcus*, which corresponded to the results of the alpha diversity analysis.

### 3.6. Preliminary analysis of the correlation between the microbiota and characteristic index

**Figure 4:**
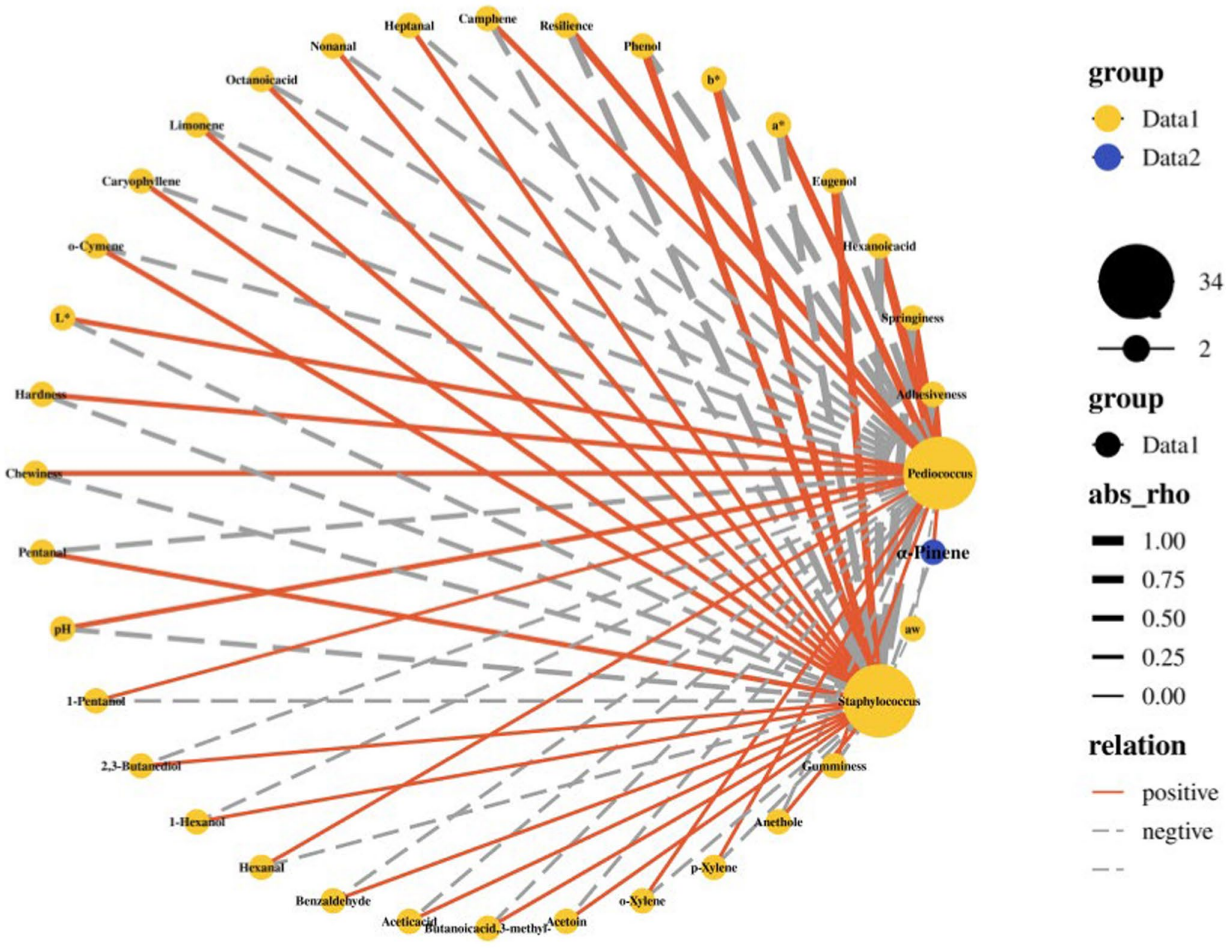
Pearson correlation analysis between bacterial community and various indexes in fermented sausage at 8 days. The edge width is proportional to the correlation strength. The solid line represents a positive correlation between the two indicators, while the dotted line represents a negative correlation between the two indicators.

To determine the effects of different amounts of cranberry powder on the physical characteristics and microbial community of samples at 8 days, the correlation between basic indicators (a_w_, pH, L* value, a* value, b* value, Texture profile analysis (TPA) and some major volatile flavor compounds) and the relative abundance of the major two genera (*Staphylococcus*, *Pediococcus*) was analyzed (Figure 4 and Supplementary Table 5). It was shown that pH, L*, hardness and chewiness had a negative correlation with *Staphylococcus* (Pearson’s correlation coefficient *r* = −0.4, *p* = 0.75) and a positive correlation with *Pediococcus* (Pearson’s correlation coefficient *r* = 0.4, *p* = 0.75). At the same time, springiness and adhesiveness had a strong positive correlation with *Pediococcus* (Pearson’s correlation coefficient *r* = 1, *p* = 0.083) and a negative correlation with *Staphylococcus* (Pearson’s correlation coefficient *r* = −1, *p* = 0.083). Moreover, hexanoic acid had a strong positive correlation with *Pediococcus* (Pearson’s correlation coefficient *r* = 0.95, *p* = 0.05) and a negative correlation with *Staphylococcus* (Pearson’s correlation coefficient *r* = −0.95, *p* = 0.05). *Pediococcus* had a positive correlation with 1-pentanol (Pearson’s correlation coefficient *r* = 0.52, *p* = 0.33), a* and resilience (Pearson’s correlation coefficient *r* = 0.8, *p* = 0.33) and then had a negative correlation with phenol and eugenol (Pearson’s correlation coefficient *r* = −0.8, *p* = 0.33). However, the correlation between these four indicators (a*, resilience, phenol and eugenol) and *Staphylococcus* was the opposite. 1-Hexanol, benzaldehyde, acetic acid, acetoin and 2,3-butanediol had a positive correlation with *Staphylococcus* (Pearson’s correlation coefficient *r* = 0.2, *p* = 0.92) and a negative correlation with *Pediococcus* (Pearson’s correlation coefficient *r* = −0.2, *p* = 0.92).

## 4. Discussion

This study mainly focused on the effects of adding different amounts of cranberry powder on the quality characteristics of fermented sausage. We consistently found that the proper content (5 g/kg) of cranberry powder could effectively promote the growth of *Staphylococcus*. Relevant studies found that the nitrate reductase activity of *Staphylococcus* was closely related to its growth, while the increase in bacteria also enhanced the reductase activity (30). The nitrate reductase activity of *Staphylococcus* could directly affect the process of the interaction between myoglobin and NO to form nitrosomyoglobin (16), which could result in the fermented sausages obtaining an ideal color. This may also be one of the reasons why the sausage that was fermented with 5 g/kg cranberry powder had a better L*, a*, and b* values. Additionally, adding cranberry powder to fermented sausage could increase the safety of the fermented sausage products because the powder could effectively inhibit the growth of spoilage bacteria. Therefore, cranberry powder could play the same role (color protection and bacteriostatic) as nitrite in fermented sausage and could be used as a natural substitute to replace the application of nitrite in fermented sausage.

The flavor compounds in the fermented sausages originated not only from the addition of spices but also from the reactions that produces volatile compounds in fermented sausage, and these reactions can be divided into chemical reactions and microbial metabolic reactions (31). We surprisingly found that the addition of cranberry powder was helpful and increased the contents of terpenes in each group, such as γ-terpinene and caryophyllene, which might be released mainly from black pepper (32). Furthermore, microbial metabolic reactions can be further divided into carbohydrate fermentation reactions, amino acid degradation reactions, β-oxidation reactions and staphylococcal esterase enzymatic hydrolysis (9). Correlation analysis showed that the growth of Staphylococcus in fermented sausages had a positive effect on the formation of flavor compounds such as 2,3-butanediol, acetic acid, acetoin, pentanal, heptanal, nonanal, octanoic acid, limonene, caryophyllene, o-cymene, eugenol and phenol in fermented sausage products. *Staphylococcus* plays an important role in the formation of the flavor of fermented sausage because the growth and metabolism of *Staphylococcus* can transform carbohydrates, amino acids and fatty acids in meat (33). In addition, the proper addition of cranberry powder effectively increased the relative abundance of *Staphylococcus*, which was also one of the reasons why there were more flavor compounds in the M5 samples than in the other samples. According to the analysis mentioned above, acetoin was the main flavor component in the four groups at the end of fermentation (4 days) and the finished product stage (8 days). Ketones are usually produced from the Maillard reaction. However, the sausages were not produced through smoking or baking, and several ketone compounds detected in this study should be produced by lipid oxidation and enzymatic hydrolysis of staphylococcal esterase (34, 35). The reason for this phenomenon was that the antioxidant capacity of cranberry powder could inhibit the metabolism of microorganisms and the occurrence of oxidation reactions in samples. Furthermore, we found that the higher relative abundance of Staphylococcus in the M5 group promoted the accumulation of acein, which was also found by the analysis of alpha diversity.

Normally, carbohydrate metabolism in fermented sausage is mainly carried out by lactic acid bacteria. Carbohydrates in fermented sausage are metabolized into lactic acid, acetic acid and other organic acids that decrease the pH value of fermented sausage and denaturate meat protein, thus effectively improving the texture characteristics of sausage (36, 37). In our study, *Pediococcus* bacteria were considered responsible for improving the texture characteristics of fermented sausage because they could decompose carbohydrate and protein into lactic acid, amino acid and other organic acids, which could produce fibrin gelatinize to improve the hardness and cohesion of the final product (38). Some studies have shown that when the pH value of the system was far from the isoelectric point of the protein, the water-holding capacity of myosin gel gradually increased, but a lower pH value caused muscle proteins to undergo hydrolysis (39, 40). Although the relative abundance of *Pediococcus* in the M5 samples (8 days) was lower than that in the other groups, the fermented sausage in the M5 group had better texture characteristics due to its relatively high pH value.

## 5. Conclusion

In this study, we investigated the effects of different amounts of cranberry powder on the quality characteristics and microbial diversity of fermented sausage. We revealed that the addition of 5 g/kg cranberry powder could result in better quality characteristics (color, texture and flavor, etc.) in fermented sausage and effectively inhibited the growth of spoilage microorganisms. Furthermore, we found that Staphylococcus had a positive effect on the formation of flavor and Pediococcus had a positive effect on the texture characteristics of fermented sausage products. In conclusion, our study provided a new idea for nitrite substitution in fermented sausage, which provided a new theoretical basis for producing fermented sausage in regard to its safety and quality.

## Data availability statement

The raw 16S rDNA sequence data reported in this paper have been deposited in online repositories. The names of the repository/repositories and accession number(s) can be found below: https://www.ncbi.nlm.nih.gov/sra/PRJNA952006 (SRA). Other datasets generated or analyzed during this study are included in this article/Supplementary material.

## Author contributions

LK, XZ, and YS: data collection and curation. TL, XG: formal analysis and software. LY and XY: visualization, writing original draft, and writing review and editing. YD: writing review and editing. All authors contributed to the article and approved the submitted version.

## Funding

This work was supported by Food Science and Engineering College Science and Technology Plan Project of Inner Mongolia Agricultural University, China (SPKJ201904), Inner Mongolia Natural Science Foundation Program of China (No. 2021MS03012) and Food Science and Engineering College Science and Technology Plan Project of Inner Mongolia Agricultural University, China (SPKJ202011).

## Conflict of interest

The authors declare that the research was conducted in the absence of any commercial or financial relationships that could be construed as a potential conflict of interest.

## Publisher’s note

All claims expressed in this article are solely those of the authors and do not necessarily represent those of their affiliated organizations, or those of the publisher, the editors and the reviewers. Any product that may be evaluated in this article, or claim that may be made by its manufacturer, is not guaranteed or endorsed by the publisher.
