# Supplementary material for: Effects of cranberry powder on the diversity of microbial communities and quality characteristics of fermented sausage
Source: Front Nutr. 2023 Apr 11;10:1123627. doi: 10.3389/fnut.2023.1123627 (PMC10126671; doi:10.3389/fnut.2023.1123627)
Supplement: Supplementary file 1 [file Data_Sheet_1.docx]

Supplementary Material

# Supplementary Table

# Supplementary Table 1. Volatile flavour compounds identified and quantified (µg/kg) in Group M3, M5, M7 and CK fermented sausages by GC–MS during processing period.

| Flavor Compounds | RT | Formula | 0d | | | | 1d | | | | 4d | | | | 8d | | | |
| --- | --- | --- | --- | --- | --- | --- | --- | --- | --- | --- | --- | --- | --- | --- | --- | --- | --- | --- |
|  |  |  | M3 | M5 | M7 | CK | M3 | M5 | M7 | CK | M3 | M5 | M7 | CK | M3 | M5 | M7 | CK |
| Alcohols |  |  |  |  |  |  |  |  |  |  |  |  |  |  |  |  |  |  |
| Ethanol | 1.35 | C2H6O | 5.05±1.87ab | 1.93±1.57b | 2.66±0.71b | 13.37±10.2a | 3.86±1.67a | 2.6±0.92a | 3.16±0.49a | 2.96±0.24a | nd. | 1.13±0.22 | — | 1.21±0.35 | 2.03±0.87 | 1.74±0.31 | nd. | 2.41±0.58 |
| 1-Butanol,3-methyl- | 4.02 | C5H12O | nd. | nd. | nd. | nd. | nd. | nd. | nd. | nd. | nd. | nd. | nd. | nd. | 0.53±0.16a | 0.63±0.09a | 0.71±0.26a | 0.57±0.15a |
| 1-Pentanol | 4.84 | C5H12O | 0.75±0.22a | 0.93±0.46a | 0.7±0.29a | 0.66±0.31a | 0.56±0.04a | 0.78±0.41a | 0.89±0.49a | 1.06±0.51a | nd. | nd. | 1.53±0.17 | 0.92±0.16 | 0.56±0.19b | 1.19±0.13a | 0.71±0.26b | 0.58±0.1b |
| 2,3-Butanediol | 5.35 | C4H10O2 | nd. | nd. | nd. | nd. | nd. | nd. | nd. | nd. | 4.15±2.48ab | 4.65±1.31ab | 6.15±0.95a | 1.65±1.2b | 6.82±2.04a | 8.34±1.35a | 13.01±8.55a | 5.34±1.65a |
| 1-Hexanol | 7.71 | C6H14O | nd. | 0.25±0.12 | nd. | nd. | nd. | 0.7±0.46 | 1.79±0.89 | 2.31±1.48 | 2.63±1.52ab | 0.94±0.11c | 2.99±0.19a | 1.36±0.3bc | 0.35±0.09c | 0.85±0.12b | 1.08±0.11a | 0.32±0.02c |
| 2-Butanol,3-methyl- | 8.19 | C5H12O | nd. | nd. | nd. | nd. | nd. | nd. | nd. | nd. | nd. | nd. | nd. | nd. | nd. | 0.53±0.7 | 0.43±0.37 | nd. |
| 2-Heptanol | 8.55 | C7H16O | 0.32±0.15 | nd. | 0.31±0.25 | 0.53±0.47 | 0.1±0.07b | 0.09±0.06b | 0.3±0.13a | 0.2±0.08ab | 0.14±0.07 | 0.33±0.24 | nd. | 0.16±0.07 | 0.19±0.08a | 0.18±0.02a | 0.16±0.07a | 0.19±0.1a |
| 1-Octen-3-ol | 10.89 | C8H16O | nd. | 0.28±0.19 | 0.13±0.06 | 0.27±0.14 | nd. | nd. | nd. | nd. | nd. | nd. | nd. | nd. | nd. | nd. | nd. | nd. |
| Eucalyptol | 12.25 | C10H18O | 3.96±4.78a | 0.62±0.69a | 0.58±0.33a | 0.75±0.69a | 0.65±0.76a | 0.86±0.21a | 1.2±0.1a | 0.86±0.8a | 0.58±0.45a | 0.37±0.06a | 6.27±9.37a | 0.27±0.14a | 0.51±0.28a | 0.49±0.52a | 0.85±0.5a | 1.25±0.08a |
| 1-Octanol | 13.41 | C8H18O | nd. | nd. | nd. | nd. | nd. | 0.38±0.19 | nd. | nd. | nd. | nd. | nd. | nd. | nd. | 0.48±0.13 | nd. | nd. |
| 1,6-Octadien-3-ol,3,7-dimethyl- | 14.16 | C10H18O | 0.81±0.37a | 2.09±1.35a | 1.55±0.83a | 1.77±0.98a | 3.32±0.81a | nd. | nd. | nd. | nd. | nd. | nd. | nd. | 2.51±0.84a | 2.99±0.07a | 3.43±1.14a | 2.7±0.07a |
| endo-Borneol | 16.16 | C10H18O | 0.54±0.07a | 0.98±0.63a | 0.74±0.4a | 0.92±0.52a | 1.2±0.34a | 1.03±0.24a | 1.43±0.35a | 1.26±0.36a | 1.27±0.34a | 0.97±0.07a | 1.03±0.15a | 0.99±0.1a | 0.89±0.28a | 1.09±0.1a | 1.29±0.54a | 1.14±0.06a |
| Terpinen-4-ol | 16.33 | C10H18O | 0.58±0.46a | 0.46±0.28a | 0.34±0.17a | 0.41±0.22a | 0.62±0.15a | 0.47±0.12a | 0.65±0.19a | 0.62±0.17a | 0.7±0.2a | 0.51±0.06ab | 0.5±0.08ab | 0.44±0.01b | 0.53±0.19a | 0.54±0.03a | 0.62±0.2a | 0.53±0.04a |
| α-Terpineol | 16.73 | C10H18O | 0.49±0.09a | 0.9±0.63a | 0.59±0.31a | 0.74±0.41a | 1.12±0.3a | 0.91±0.23a | 1.26±0.37a | 1.08±0.26a | nd. | 0.89±0.12 | 0.89±0.15 | 0.78±0.03 | 0.9±0.29a | 0.94±0.04a | 1.15±0.39a | 0.92±0.08a |
| 2,6-Octadien-1-ol,3,7-dimethyl-,(Z)- | 16.92 | C10H18O | 0.2±0.15a | 0.63±0.54a | 0.3±0.16a | 0.4±0.21a | 0.82±0.19a | nd. | nd. | nd. | nd. | nd. | nd. | nd. | 0.51±0.16a | 0.54±0.03a | 0.65±0.2a | 0.52±0.04a |
|  |  |  |  |  |  |  |  |  |  |  |  |  |  |  |  |  |  |  |
| Aldehydes |  |  |  |  |  |  |  |  |  |  |  |  |  |  |  |  |  |  |
| Pentanal | 3.21 | C5H10O | nd. | 0.41±0.18 | nd. | nd. | 1.64±0.37a | 2.32±1.55a | 1.94±1.17a | 2.03±0.61a | 0.67±0.16c | 2.07±0.63b | 3.22±0.48a | 2.32±0.66ab | 1.97±0.69a | 3.45±0.44a | 3.47±1.84a | 2.77±0.65a |
| Hexanal | 5.71 | C6H12O | 0.37±0.04a | 1.25±0.69a | 1.05±0.55a | 0.7±0.31a | 10.57±1.7a | 4.84±3.41a | 4.11±4.5a | 5.78±2.86a | 5.7±3.01b | 7.65±2.56b | 13.99±2.7a | 7.09±4.33b | 1.99±0.64b | 9.84±0.35a | 8.1±0.81c | 2.07±0.34c |
| Heptanal | 8.66 | C7H14O | 0.48±0.63a | 0.48±0.26a | 0.55±0.32a | 0.21±0.09a | 4±0.52a | 1.59±1.96a | nd. | 2.99±0.84a | nd. | nd. | nd. | nd. | 1.98±0.61a | 2.53±2.03a | 3.64±0.32a | 2.43±0.19a |
| Benzaldehyde | 10.57 | C7H6O | nd. | 0.79±0.6 | nd. | nd. | 6.41±3.36a | 5.21±1.63a | 6.08±2.89a | 3.53±1.3a | 13.28±9.91a | 4.3±0.33ab | 6.21±1.37ab | 3.14±0.94b | 2.53±0.71a | 2.75±0.72a | 2.92±1.39a | 2.46±0.55a |
| Benzeneacetaldehyde | 12.87 | C8H8O | nd. | nd. | nd. | nd. | 0.32±0.03a | 0.36±0.17a | 0.43±0.23a | 0.37±0.04a | 0.81±0.31 | nd. | 0.57±0.22 | 0.46±0.11 | 0.67±0.17ab | 0.9±0.08a | 0.8±0.23ab | 0.58±0.04b |
| Nonanal | 14.16 | C9H18O | 1.05±1.01 | nd. | 0.83±0.4 | 0.71±0.34 | 4.97±0.66a | 4.12±2.06a | 4.44±3.59a | 4.58±1.23a | 8.79±7.02a | 3.77±1.04a | 8.7±1.47a | 4.37±1.61a | 5.54±1.95a | 4.34±0.84a | 4.46±0.62a | 5.65±1.47a |
| Decanal | 16.91 | C10H20O | nd. | nd. | nd. | nd. | nd. | 0.3±0.11 | nd. | nd. | nd. | nd. | 0.38±0.06 | 0.26±0.03 | nd. | 0.32±0.06 | nd. | nd. |
| 2-Decenal,(E)- | 18.32 | C10H18O | nd. | nd. | nd. | nd. | 0.44±0.08 | nd. | nd. | nd. | nd. | nd. | nd. | nd. | nd. | 0.3±0.09 | 0.31±0.07 | nd. |
|  |  |  |  |  |  |  |  |  |  |  |  |  |  |  |  |  |  |  |
| Acids |  |  |  |  |  |  |  |  |  |  |  |  |  |  |  |  |  |  |
| Ala-Gly | 1.17 | C5H10N2O3 | 1.59±0.58a | 1.51±0.93a | 1.52±0.91a | 1.09±0.42a | 1.89±0.68a | 1.35±0.37a | 1.46±0.31a | 1.52±0.43a | 2.16±0.45a | 1.57±0.52a | 1.78±0.05a | 0.48±0.36b | 0.98±0.81a | 1.54±0.56a | 1.7±0.59a | 1.82±0.26a |
| Glycine | 1.6 | C2H5NO2 | nd. | nd. | nd. | nd. | nd. | nd. | nd. | 5.59±3.05 | nd. | 11.44±2.42 | 7.32±1.05 | 3.78±1.47 | 11.45±4.79 | nd. | nd. | 8.07±3.11 |
| Aceticacid | 1.86 | C2H4O2 | nd. | nd. | nd. | nd. | 12.13±6.28 | nd. | nd. | nd. | nd. | nd. | nd. | nd. | 39.18±10.54a | 48.83±5.6a | 57.67±28.98a | 29.05±8.95a |
| Propanoicacid,2-methyl- | 4.48 | C4H8O2 | nd. | nd. | nd. | nd. | nd. | nd. | nd. | nd. | nd. | nd. | nd. | nd. | 0.37±0.13 | nd. | 0.76±0.46 | 0.25±0.09 |
| Butanoicacid,3-methyl- | 6.93 | C5H10O2 | nd. | nd. | nd. | nd. | 1.02±0.29 | nd. | nd. | nd. | nd. | nd. | nd. | nd. | 2.31±0.8a | 3.04±1.21a | 3.54±2.1a | 1.37±0.46a |
| Butanoicacid,2-methyl- | 7.15 | C5H10O2 | nd. | nd. | nd. | nd. | nd. | nd. | nd. | nd. | nd. | nd. | nd. | nd. | 0.72±0.24a | 0.67±0.13a | 0.79±0.35a | 0.53±0.11a |
| Hexanoicacid | 10.9 | C6H12O2 | nd. | nd. | nd. | nd. | 1.98±0.43 | nd. | nd. | nd. | nd. | nd. | nd. | nd. | 1.35±1.15a | 2.43±0.41a | 1.34±0.99a | 1.35±0.05a |
| Pentanoicacid | 10.92 | C5H10O2 | nd. | nd. | nd. | nd. | nd. | nd. | nd. | nd. | nd. | nd. | nd. | nd. | nd. | 1.56±1.11 | nd. | nd. |
| Octanoicacid | 15.99 | C8H16O2 | nd. | nd. | nd. | nd. | 1.98±0.69 | nd. | nd. | nd. | nd. | nd. | nd. | nd. | 2.73±0.96a | 2.25±0.62a | 3.66±2.44a | 2.15±0.33a |
|  |  |  |  |  |  |  |  |  |  |  |  |  |  |  |  |  |  |  |
| Esters |  |  |  |  |  |  |  |  |  |  |  |  |  |  |  |  |  |  |
| Propanoicacid,2-hydroxy-,ethylester,(S)- | 5.99 | C5H10O3 | nd. | nd. | nd. | nd. | nd. | nd. | nd. | nd. | nd. | nd. | nd. | nd. | 0.74±0.24 | 0.67±0.08 | nd. | 1.02±0.13 |
| 2-Heptanol,acetate | 12.29 | C9H18O2 | 0.36±0.33a | 0.57±0.73a | 0.18±0.09a | 0.22±0.1a | 0.51±0.07 | nd. | nd. | nd. | nd. | nd. | nd. | nd. | nd. | nd. | nd. | nd. |
| Octanoicacid,methylester | 14.55 | C9H18O2 | 0.55±0.56a | 0.48±0.29a | 0.33±0.15a | 0.27±0.11a | nd. | nd. | nd. | nd. | nd. | nd. | nd. | nd. | nd. | nd. | nd. | nd. |
| Octanoicacid,ethylester | 16.59 | C10H20O2 | nd. | nd. | nd. | 1.69±0.93 | nd. | nd. | nd. | nd. | nd. | nd. | nd. | nd. | 0.69±0.27 | 0.69±0.15 | nd. | 0.72±0.13 |
| Bornylacetate | 18.78 | C12H20O2 | nd. | 0.27±0.18 | 0.22±0.1 | 0.15±0.06 | 0.49±0.14 | nd. | nd. | nd. | nd. | nd. | nd. | nd. | 0.35±0.13a | 0.3±0.02a | 0.45±0.18a | 0.32±0.04a |
|  |  |  |  |  |  |  |  |  |  |  |  |  |  |  |  |  |  |  |
| Phenols |  |  |  |  |  |  |  |  |  |  |  |  |  |  |  |  |  |  |
| Phenol | 3.05 | C6H6O | nd. | nd. | nd. | nd. | nd. | nd. | nd. | nd. | 2.46±0.35 | 1.72±0.46 | 1.91±0.58 | nd. | 1.39±0.48a | 1.47±0.08a | 1.92±0.93a | 1.55±0.43a |
| Eugenol | 20.47 | C10H12O2 | 5.7±7.25a | 1.31±1.17a | 0.66±0.42a | 0.97±0.57a | 1.68±0.46a | 1.42±0.4a | 2.02±0.54a | 1.63±0.68a | 1.81±0.64a | 1.19±0.17ab | 1.01±0.22b | 0.97±0.08b | 1.28±0.39a | 1.12±0.03a | 1.36±0.52a | 1.18±0.17a |
|  |  |  |  |  |  |  |  |  |  |  |  |  |  |  |  |  |  |  |
| Ketones |  |  |  |  |  |  |  |  |  |  |  |  |  |  |  |  |  |  |
| Acetoin | 3.43 | C4H8O2 | nd. | nd. | nd. | nd. | 17.31±5.4 | 17.57±5.64 | nd. | 11.37±1.52 | 46.99±6.51b | 64.58±2.37a | 45.66±9.3b | 11.09±0.36c | 61.17±20.85a | 65.94±6.06a | 90.13±51.91a | 35.55±18.09a |
| 5-Hepten-2-one,6-methyl- | 11.05 | C8H14O | nd. | 1.84±0.89 | 1.39±0.59 | 1.91±0.88 | nd. | nd. | nd. | nd. | nd. | nd. | nd. | nd. | nd. | nd. | nd. | nd. |
| Camphor | 15.54 | C10H16O | nd. | 0.37±0.22 | 0.3±0.16 | 0.34±0.2 | 0.58±0.15 | nd. | 0.45±0.15 | 0.48±0.04 | 0.56±0.14a | 0.43±0.05ab | 0.48±0.04ab | 0.37±0.03b | 0.43±0.14a | 0.44±0.02a | 0.49±0.16a | 0.45±0.05a |
|  |  |  |  |  |  |  |  |  |  |  |  |  |  |  |  |  |  |  |
| Terpenes |  |  |  |  |  |  |  |  |  |  |  |  |  |  |  |  |  |  |
| 1,3,5-Cycloheptatriene | 4.71 | C7H8 | nd. | 0.35±0.13 | 0.27±0.09 | 0.32±0.18 | nd. | 0.54±0.25 | 0.45±0.2 | 0.56±0.15 | nd. | nd. | nd. | nd. | nd. | nd. | nd. | nd. |
| (1R)-2,6,6-Trimethylbicyclo[3.1.1]hept-2-ene | 8.66 | C10H16 | 1.23±0.84 | nd. | nd. | 0.34±0.2 | 0.92±0.2 | nd. | nd. | nd. | nd. | nd. | nd. | nd. | 0.91±0.21a | 1.22±0.23a | 0.88±0.62a | 1.02±0.15a |
| α-Pinene | 9.24 | C10H16 | 0.83±0.96a | 0.22±0.08a | 0.23±0.1a | 0.34±0.2a | 0.92±0.2 | nd. | nd. | 4.05±4.62 | 3.81±4.55a | 1.04±0.23a | 1.2±0.05a | 1.05±0.23a | 0.91±0.21a | 4.78±6.01a | 1.07±0.36a | 1.02±0.15a |
| Camphene | 9.56 | C10H16 | 1.35±1.64a | 0.33±0.11a | 0.31±0.16a | 0.34±0.15a | 1.05±0.26a | 0.47±0.21b | 0.78±0.36ab | 0.95±0.15ab | 1.1±0.33a | 1.14±0.26a | 1.2±0.26a | 1.32±0.45a | 1.19±0.4a | 1.12±0.23a | 1.1±0.58a | 1.1±0.19a |
| β-Pinene | 10.6 | C10H16 | 0.22±0.18a | 0.22±0.17a | 0.42±0.18a | 0.47±0.23a | 1±0.16 | nd. | nd. | 2.48±1.67 | nd. | 1.21±0.13 | 1.11±0.08 | 0.88±0.02 | 0.88±0.3a | 1.13±0.17a | 0.55±0.45a | 1.07±0.26a |
| Bicyclo[3.1.1]heptane,6,6-dimethyl-2-methylene-,(1S)- | 10.64 | C10H16 | 0.41±0.14 | nd. | nd. | nd. | 2.01±1.77 | nd. | nd. | nd. | nd. | nd. | nd. | nd. | nd. | 1.93±3.31 | nd. | nd. |
| 3-Carene | 10.89 | C10H16 | 0.95±0.88b | 1.52±1.04ab | 2.95±1.26a | 0.34±0.2b | 3.83±4.86 | nd. | 3.64±2.61 | 10.04±8.58 | 3.81±4.55 | 1.04±0.23 | nd. | 3.54±4.44 | 0.91±0.21a | 1.22±0.23a | 3.49±3.98a | 1.02±0.15a |
| β-Myrcene | 11.03 | C10H16 | nd. | nd. | nd. | nd. | 4.11±0.5 | nd. | nd. | 3.96±2.75 | nd. | 4.69±0.77 | 4.85±0.48 | nd. | 3.5±3.32 | nd. | 1.87±2.99 | nd. |
| β-Ocimene | 11.56 | C10H16 | nd. | nd. | 2.95±1.26 | 3.35±1.62 | 11.11±1.49 | nd. | nd. | nd. | nd. | nd. | nd. | nd. | nd. | 11.6±0.64 | nd. | 10.37±1.4 |
| D-Limonene | 12.18 | C10H16 | nd. | nd. | nd. | nd. | 14.27±1.72 | 7.32±4.54 | 9.8±3.98 | nd. | 16.66±3.83a | 16.83±2.4a | 15.02±1.13ab | 11.76±0.54b | 13.88±4.78a | 15.89±1.62a | 16.47±5.6a | 14.8±1.91a |
| Limonene | 12.19 | C10H16 | 5.94±2.98a | 5.1±1.68a | 5.31±2.04a | 6.82±3.42a | 14.27±1.72a | 7.32±4.54a | 9.8±3.98a | 19.13±11.55a | 16.66±3.83a | 16.83±2.4a | 15.02±1.13ab | 11.76±0.54b | 13.88±4.78a | 15.89±1.62a | 16.47±5.6a | 14.8±1.91a |
| Bicyclo[3.1.0]hexane,4-methylene-1-(1-methylethyl)- | 12.25 | C10H16 | nd. | 4.85±1.55 | 5.17±5.18 | nd. | nd. | nd. | nd. | nd. | nd. | nd. | nd. | nd. | 12.1±10.85a | 1.13±0.17a | 9.37±14.83a | 1.07±0.26a |
| γ-Terpinene | 13 | C10H16 | nd. | 0.38±0.23 | 0.3±0.16 | 0.35±0.16 | 0.71±0.08 | nd. | 0.51±0.2 | 0.91±0.52 | 0.84±0.19a | 0.79±0.08ab | 0.78±0.05ab | 0.61±0.03b | 0.7±0.22a | 0.82±0.07a | 0.8±0.26a | 0.73±0.09a |
| Cyclohexene,1-methyl-4-(1-methylethylidene)- | 13.01 | C10H16 | 0.25±0.31a | 0.16±0.1a | 0.3±0.16a | 0.28±0.22a | 5.74±8.77a | nd. | nd. | nd. | nd. | nd. | nd. | nd. | 0.59±0.43a | 0.41±0.32a | 0.54±0.57a | 0.33±0.21a |
| 2-Carene | 13.01 | C10H16 | 0.26±0.3a | 0.13±0.05a | 0.24±0.1a | 0.15±0.12a | 0.55±0.33 | 0.36±0.16 | 0.33±0.3 | nd. | nd. | nd. | nd. | nd. | 0.18±0.06 | nd. | nd. | 0.37±0.34 |
| Copaene | 20.48 | C15H24 | 0.91±0.56a | 0.42±0.16a | 0.73±0.38a | 0.85±0.44a | 1.57±0.28a | 0.97±0.36a | 1.39±0.55a | 1.49±0.73a | 1.65±0.61a | 1.06±0.14a | 1.17±0.23a | 1.09±0.14a | 1.25±0.39a | 1.18±0.13a | 1.55±0.5a | 1.22±0.18a |
| Caryophyllene | 21.21 | C15H24 | 1.02±0.58a | 2.96±2.51a | 1.97±0.98a | 2.41±1.17a | 4.18±0.98a | 2.67±0.88a | 3.69±1.34a | 4.27±1.9a | 4.74±1.63a | 3.08±0.42ab | 3.12±0.49ab | 2.83±0.14b | 3.13±0.94a | 3.37±0.47a | 3.97±1.12a | 3.25±0.46a |
| Bicyclo[7.2.0]undec-4-ene,4,11,11-trimethyl-8-methylene-,[1R-(1R*,4Z,9S*)]- | 21.21 | C15H24 | 1.02±0.58a | 2.96±2.51a | 1.97±0.98a | 2.41±1.17a | nd. | nd. | nd. | nd. | nd. | nd. | nd. | nd. | 3.13±0.94a | 3.37±0.47a | 3.97±1.12a | 3.25±0.46a |
| Cedrene | 21.84 | C15H24 | 1.7±1.8 | 0.44±0.38 | 0.1±0.04 | nd. | nd. | nd. | nd. | nd. | nd. | nd. | nd. | nd. | 0.64±0.5 | 0.53±0.44 | nd. | 0.86±0.22 |
| Benzene,1-(1,5-dimethyl-4-hexenyl)-4-methyl- | 21.84 | C15H22 | 1.63±1.84a | 0.88±0.72a | 0.48±0.22a | 0.58±0.31a | 1±0.23 | nd. | nd. | nd. | nd. | nd. | nd. | nd. | 0.65±0.14a | 0.61±0.04a | 0.86±0.4a | 0.64±0.08a |
| Humulene | 22.65 | C15H24 | nd. | 0.16±0.04 | 0.19±0.1 | 0.21±0.13 | 0.35±0.07a | 0.27±0.08a | 0.35±0.13a | 0.2±0.18a | 0.32±0.18a | 0.28±0.03a | 0.27±0.02a | 0.19±0.02a | 0.26±0.07a | 0.26±0.02a | 0.33±0.12a | 0.26±0.04a |
| 1,3-Cyclohexadiene,5-(1,5-dimethyl-4-hexenyl)-2-methyl-,[S-(R*,S*)]- | 23.49 | C15H24 | nd. | nd. | 2.36±1.21 | nd. | 4.71±1.1 | nd. | nd. | nd. | nd. | nd. | nd. | nd. | 1.97±1.6 | 2.53±0.23 | 3.46±1.13 | nd. |
| α-Farnesene | 23.63 | C15H24 | nd. | 0.71±0.43 | 0.76±0.38 | nd. | 1.35±0.3 | 0.95±0.34 | nd. | 1.14±0.41 | 1.4±0.71a | 0.69±0.17a | 0.84±0.18a | 0.73±0.04a | nd. | 0.83±0.06 | 1.13±0.29 | 0.94±0.16 |
| β-Bisabolene | 23.69 | C15H24 | 0.28±0.08a | 0.82±0.64a | 0.51±0.26a | 0.57±0.3a | 0.92±0.22a | 0.67±0.24a | 0.91±0.29a | 0.79±0.28a | 0.95±0.5a | 0.5±0.08a | 0.56±0.12a | 0.58±0.11a | 0.64±0.14a | 0.55±0.04a | 0.76±0.25a | 0.6±0.12a |
| Naphthalene,1,2,3,5,6,8a-hexahydro-4,7-dimethyl-1-(1-methylethyl)-,(1S-cis)- | 23.69 | C15H24 | 0.1±0.04 | 0.32±0.28 | 0.13±0.07 | nd. | 0.19±0.07a | nd. | nd. | nd. | nd. | nd. | nd. | nd. | 0.11±0.05a | 0.26±0.27a | 0.16±0.05a | 0.12±0.04a |
| Cyclohexene,3-(1,5-dimethyl-4-hexenyl)-6-methylene-,[S-(R*,S*)]- | 23.75 | C15H24 | 0.64±0.31a | 1.21±0.97a | 0.73±0.36a | 0.74±0.37a | 1.3±0.31 | nd. | nd. | nd. | nd. | nd. | nd. | nd. | 0.88±0.15a | 0.52±0.37a | 1.02±0.36a | 0.86±0.22a |
| 1H-3a,7-Methanoazulene,octahydro-3,8,8-trimethyl-6-methylene-,[3R-(3α,3aβ,7β,8aα)]- | 23.75 | C15H24 | 0.64±0.31 | 1.21±0.97 | 0.73±0.36 | nd. | nd. | nd. | nd. | nd. | nd. | nd. | nd. | nd. | 0.88±0.15a | 0.75±0.06a | 1.02±0.36a | 0.86±0.22a |
|  |  |  |  |  |  |  |  |  |  |  |  |  |  |  |  |  |  |  |
| Other |  |  |  |  |  |  |  |  |  |  |  |  |  |  |  |  |  |  |
| Toluene | 3.53 | C7H8 | 0.49±0.39 | 0.35±0.13 | nd. | 0.32±0.18 | 0.53±0.12 | 0.54±0.25 | 0.45±0.2 | nd. | 0.7±0.18a | 0.72±0.13a | 0.72±0.05a | 0.41±0.02b | 0.38±0.12a | 0.5±0.09a | 0.46±0.16a | 0.45±0.06a |
| Ethylbenzene | 6.38 | C8H10 | 0.15±0.1a | 0.31±0.12a | 0.42±0.32a | 0.5±0.13a | 0.97±0.18a | 0.74±0.48a | 0.65±0.45a | 0.65±0.28a | 0.76±0.15b | 0.74±0.08b | 1.02±0.09a | 0.54±0.04c | 0.35±0.09a | 0.52±0.02a | 0.5±0.16a | 0.45±0.07a |
| o-Xylene | 7.33 | C8H10 | 0.62±0.08a | 0.73±0.25a | 0.54±0.21a | 0.44±0.28a | nd. | nd. | nd. | nd. | 1.28±0.99 | nd. | nd. | 0.99±0.76 | 0.58±0.8a | 1.64±0.08a | 1.04±0.98a | 0.95±0.8a |
| p-Xylene | 7.61 | C8H10 | 0.84±0.79a | 0.73±0.25a | 0.6±0.2a | 1.07±0.67a | nd. | 2.4±1.73 | 1.05±0.73 | 1.66±0.71 | 2.08±0.55b | 2.57±0.47ab | 3.49±0.99a | 1.52±0.11b | 1.14±0.35a | 1.64±0.08a | 1.47±0.55a | 1.37±0.22a |
| o-Cymene | 11.57 | C10H14 | 2.1±2.36a | 0.7±0.34a | 0.63±0.27a | 0.79±0.39a | 1.58±0.26a | 0.87±0.43a | 1.18±0.4a | 2.12±1.22a | 1.75±0.36a | 1.73±0.21a | 1.54±0.1ab | 1.29±0.05b | 1.49±0.55a | 1.74±0.16a | 1.76±0.68a | 1.62±0.25a |
| Anethole | 17.7 | C10H12O | 0.96±0.4a | 2.48±1.82a | 7.35±7.19a | 9.7±5.11a | 17.07±3.66a | 11.31±3.5a | 17.59±5.24a | 16.89±6.38a | 16.7±3.53a | 12.39±1.87ab | 11.94±2.05ab | 7.39±6.12b | 12.79±4.07a | 12.91±1.32a | 16.07±5.36a | 12.34±1.04a |
| Estragole | 17.98 | C10H12O | 1.05±0.27 | 4.01±2.17 | 7.35±7.19 | nd. | nd. | 11.31±3.5 | 17.59±5.24 | 16.89±6.38 | nd. | 12.39±1.87 | 11.94±2.05 | 7.39±6.12 | 12.79±4.07a | 12.91±1.32a | 16.07±5.36a | 12.34±1.04a |

# Mean±SD (n=3); Different lowercase letters represent significant differences between groups at the same time (P<0.05), while the same letter indicated no significant difference(P>0.05);nd.: No flavor substance detected.

Supplementary Table 2. Relative abundance values of each component at phylum level

| phylum | CK-8d | CK-4d | CK-1d | CK-0d | M3-8d | M3-4d | M3-1d | M3-0d | M5-8d | M5-4d | M5-1d | M5-0d | M7-8d | M7-4d | M7-1d | M7-0d |
| --- | --- | --- | --- | --- | --- | --- | --- | --- | --- | --- | --- | --- | --- | --- | --- | --- |
| Firmicutes | 94.51 | 96.90 | 98.02 | 80.74 | 93.27 | 97.28 | 97.31 | 72.25 | 98.16 | 97.43 | 94.70 | 73.98 | 95.59 | 96.96 | 97.61 | 71.43 |
| Cyanobacteria | 3.87 | 2.18 | 1.39 | 13.74 | 3.47 | 0.98 | 1.06 | 17.22 | 1.31 | 1.63 | 4.52 | 15.54 | 2.69 | 1.35 | 1.77 | 13.90 |
| Proteobacteria | 1.29 | 0.75 | 0.35 | 4.40 | 2.86 | 1.56 | 1.42 | 7.35 | 0.36 | 0.74 | 0.54 | 8.55 | 1.42 | 1.36 | 0.43 | 11.69 |
| Bacteroidetes | 0.23 | 0.12 | 0.18 | 0.72 | 0.30 | 0.15 | 0.18 | 2.58 | 0.12 | 0.15 | 0.18 | 1.41 | 0.24 | 0.25 | 0.16 | 2.40 |
| Actinobacteria | 0.06 | 0.03 | 0.04 | 0.29 | 0.05 | 0.01 | 0.02 | 0.35 | 0.02 | 0.03 | 0.01 | 0.39 | 0.04 | 0.03 | 0.02 | 0.24 |
| Spirochaetes | 0.00 | 0.00 | 0.00 | 0.03 | 0.02 | 0.00 | 0.00 | 0.08 | 0.00 | 0.00 | 0.00 | 0.06 | 0.00 | 0.00 | 0.01 | 0.14 |
| Epsilonbacteraeota | 0.04 | 0.01 | 0.01 | 0.02 | 0.01 | 0.01 | 0.01 | 0.02 | 0.02 | 0.01 | 0.03 | 0.01 | 0.01 | 0.04 | 0.00 | 0.03 |
| unclassified | 0.00 | 0.00 | 0.00 | 0.01 | 0.01 | 0.00 | 0.00 | 0.05 | 0.00 | 0.00 | 0.00 | 0.01 | 0.00 | 0.00 | 0.00 | 0.04 |
| Patescibacteria | 0.00 | 0.00 | 0.01 | 0.01 | 0.01 | 0.00 | 0.00 | 0.02 | 0.00 | 0.00 | 0.00 | 0.01 | 0.00 | 0.00 | 0.00 | 0.03 |
| Kiritimatiellaeota | 0.00 | 0.00 | 0.00 | 0.01 | 0.00 | 0.00 | 0.00 | 0.02 | 0.00 | 0.00 | 0.00 | 0.01 | 0.00 | 0.00 | 0.00 | 0.04 |
| Synergistetes | 0.00 | 0.00 | 0.00 | 0.01 | 0.00 | 0.00 | 0.00 | 0.01 | 0.00 | 0.00 | 0.00 | 0.01 | 0.00 | 0.00 | 0.00 | 0.02 |
| Acidobacteria | 0.00 | 0.00 | 0.00 | 0.01 | 0.00 | 0.00 | 0.00 | 0.00 | 0.00 | 0.00 | 0.00 | 0.00 | 0.00 | 0.00 | 0.00 | 0.01 |
| Fibrobacteres | 0.00 | 0.00 | 0.00 | 0.01 | 0.00 | 0.00 | 0.00 | 0.00 | 0.00 | 0.00 | 0.00 | 0.01 | 0.00 | 0.00 | 0.00 | 0.01 |
| Planctomycetes | 0.00 | 0.00 | 0.00 | 0.01 | 0.00 | 0.00 | 0.00 | 0.01 | 0.00 | 0.00 | 0.00 | 0.00 | 0.00 | 0.00 | 0.00 | 0.00 |
| Chloroflexi | 0.00 | 0.00 | 0.00 | 0.00 | 0.00 | 0.00 | 0.00 | 0.01 | 0.00 | 0.00 | 0.00 | 0.00 | 0.00 | 0.00 | 0.00 | 0.00 |
| Verrucomicrobia | 0.00 | 0.00 | 0.00 | 0.01 | 0.00 | 0.00 | 0.00 | 0.00 | 0.00 | 0.00 | 0.00 | 0.00 | 0.00 | 0.00 | 0.00 | 0.00 |
| Deinococcus-Thermus | 0.00 | 0.00 | 0.00 | 0.00 | 0.00 | 0.00 | 0.00 | 0.00 | 0.00 | 0.00 | 0.00 | 0.00 | 0.00 | 0.00 | 0.00 | 0.00 |
| Fusobacteria | 0.00 | 0.00 | 0.00 | 0.00 | 0.00 | 0.00 | 0.00 | 0.01 | 0.00 | 0.00 | 0.00 | 0.00 | 0.00 | 0.00 | 0.00 | 0.00 |
| Lentisphaerae | 0.00 | 0.00 | 0.00 | 0.00 | 0.00 | 0.00 | 0.00 | 0.00 | 0.00 | 0.00 | 0.00 | 0.00 | 0.00 | 0.00 | 0.00 | 0.01 |

Supplementary Table 3. Relative abundance values of each component at genus level

| Genus | CK-8d | CK-4d | CK-1d | CK-0d | M3-8d | M3-4d | M3-1d | M3-0d | M5-8d | M5-4d | M5-1d | M5-0d | M7-8d | M7-4d | M7-1d | M7-0d |
| --- | --- | --- | --- | --- | --- | --- | --- | --- | --- | --- | --- | --- | --- | --- | --- | --- |
| Pediococcus | 73.39 | 62.85 | 29.72 | 60.47 | 80.90 | 37.02 | 65.09 | 59.49 | 40.67 | 54.14 | 76.16 | 64.85 | 53.66 | 57.35 | 39.59 | 58.09 |
| Staphylococcus | 20.57 | 33.63 | 67.96 | 18.92 | 10.95 | 58.25 | 31.05 | 9.50 | 55.64 | 42.63 | 18.14 | 7.04 | 41.16 | 38.71 | 57.55 | 8.90 |
| Psychrobacter | 0.30 | 0.19 | 0.09 | 1.02 | 0.88 | 0.39 | 0.44 | 2.92 | 0.07 | 0.27 | 0.15 | 2.45 | 0.42 | 0.37 | 0.17 | 3.31 |
| Acinetobacter | 0.26 | 0.18 | 0.07 | 0.75 | 0.09 | 0.03 | 0.03 | 0.62 | 0.05 | 0.10 | 0.06 | 0.82 | 0.40 | 0.44 | 0.09 | 4.06 |
| Brochothrix | 0.11 | 0.15 | 0.08 | 0.23 | 0.22 | 0.56 | 0.30 | 0.54 | 0.09 | 0.15 | 0.06 | 0.35 | 0.32 | 0.27 | 0.22 | 1.81 |
| Pseudoalteromonas | 0.09 | 0.05 | 0.02 | 0.52 | 0.13 | 0.04 | 0.04 | 0.90 | 0.02 | 0.07 | 0.04 | 1.26 | 0.23 | 0.07 | 0.02 | 0.79 |
| Macrococcus | 0.01 | 0.03 | 0.03 | 0.02 | 0.60 | 1.06 | 0.52 | 0.05 | 1.47 | 0.16 | 0.03 | 0.01 | 0.04 | 0.07 | 0.01 | 0.02 |
| Escherichia-Shigella | 0.01 | 0.01 | 0.01 | 0.07 | 1.34 | 0.86 | 0.74 | 0.45 | 0.02 | 0.02 | 0.02 | 0.11 | 0.05 | 0.04 | 0.03 | 0.24 |
| Prevotella_1 | 0.02 | 0.01 | 0.00 | 0.09 | 0.17 | 0.04 | 0.08 | 1.31 | 0.02 | 0.03 | 0.05 | 0.42 | 0.07 | 0.07 | 0.04 | 1.01 |
| Pseudomonas | 0.06 | 0.03 | 0.01 | 0.25 | 0.06 | 0.02 | 0.02 | 0.52 | 0.02 | 0.04 | 0.01 | 1.40 | 0.04 | 0.06 | 0.01 | 0.45 |
| Lactobacillus | 0.13 | 0.10 | 0.07 | 0.10 | 0.18 | 0.14 | 0.12 | 0.15 | 0.13 | 0.14 | 0.14 | 0.10 | 0.10 | 0.21 | 0.10 | 0.10 |
| Succiniclasticum | 0.02 | 0.00 | 0.00 | 0.09 | 0.11 | 0.03 | 0.03 | 0.64 | 0.01 | 0.03 | 0.02 | 0.24 | 0.05 | 0.04 | 0.02 | 0.45 |
| Mitochondria_unclassified | 0.16 | 0.05 | 0.01 | 0.30 | 0.06 | 0.02 | 0.02 | 0.30 | 0.02 | 0.04 | 0.05 | 0.29 | 0.05 | 0.03 | 0.01 | 0.26 |
| Aeromonas | 0.04 | 0.04 | 0.01 | 0.23 | 0.03 | 0.02 | 0.01 | 0.11 | 0.02 | 0.02 | 0.04 | 0.17 | 0.04 | 0.05 | 0.02 | 0.28 |
| Muribaculaceae_unclassified | 0.03 | 0.02 | 0.07 | 0.08 | 0.03 | 0.03 | 0.03 | 0.19 | 0.05 | 0.02 | 0.04 | 0.12 | 0.04 | 0.04 | 0.04 | 0.15 |
| Enterobacter | 0.04 | 0.03 | 0.02 | 0.12 | 0.03 | 0.01 | 0.01 | 0.14 | 0.03 | 0.01 | 0.04 | 0.14 | 0.02 | 0.03 | 0.01 | 0.20 |
| Paracoccus | 0.01 | 0.00 | 0.01 | 0.07 | 0.00 | 0.00 | 0.00 | 0.21 | 0.00 | 0.00 | 0.01 | 0.40 | 0.01 | 0.01 | 0.00 | 0.14 |
| Lachnospiraceae_NK3A20_group | 0.02 | 0.01 | 0.00 | 0.07 | 0.04 | 0.00 | 0.01 | 0.22 | 0.01 | 0.01 | 0.01 | 0.12 | 0.03 | 0.03 | 0.00 | 0.23 |
| others | 4.75 | 2.62 | 1.80 | 16.61 | 4.20 | 1.48 | 1.46 | 21.74 | 1.66 | 2.13 | 4.93 | 19.72 | 3.26 | 2.11 | 2.05 | 19.52 |

Supplementary Table 4. Permanova (adonis) analysis of the groups

|  | Df | SumOfSqs | R^2^ | F | Pr(>F) |
| --- | --- | --- | --- | --- | --- |
| Group | 15 | 0.48 | 0.81 | 9.05 | 0.00 |
| Residual | 32 | 0.11 | 0.19 |  |  |
| Total | 47 | 0.59 | 1 |  |  |

# Supplementary Table 5. the correlation between the microbiota and characteristic index

| Data1 | Data2 | rho | P value | relation |
| --- | --- | --- | --- | --- |
| Pediococcus | Springiness | 1 | 0.08333333 | positive |
| Adhesiveness | Pediococcus | 1 | 0.08333333 | positive |
| Springiness | Staphylococcus | -1 | 0.08333333 | negtive |
| Adhesiveness | Staphylococcus | -1 | 0.08333333 | negtive |
| Hexanoicacid | Pediococcus | 0.9486833 | 0.0513167 | positive |
| Hexanoicacid | Staphylococcus | -0.9486833 | 0.0513167 | negtive |
| Pediococcus | Phenol | -0.8 | 0.33333333 | negtive |
| Eugenol | Pediococcus | -0.8 | 0.33333333 | negtive |
| a* | Pediococcus | 0.8 | 0.33333333 | positive |
| b* | Pediococcus | -0.8 | 0.33333333 | negtive |
| Pediococcus | Resilience | 0.8 | 0.33333333 | positive |
| Phenol | Staphylococcus | 0.8 | 0.33333333 | positive |
| Eugenol | Staphylococcus | 0.8 | 0.33333333 | positive |
| a* | Staphylococcus | -0.8 | 0.33333333 | negtive |
| b* | Staphylococcus | 0.8 | 0.33333333 | positive |
| Resilience | Staphylococcus | -0.8 | 0.33333333 | negtive |
| Camphene | Pediococcus | 0.5 | 1 | positive |
| Camphene | Staphylococcus | -0.5 | 1 | negtive |
| Pediococcus | Pentanal | -0.4 | 0.75 | negtive |
| Heptanal | Pediococcus | -0.4 | 0.75 | negtive |
| Nonanal | Pediococcus | -0.4 | 0.75 | negtive |
| Octanoicacid | Pediococcus | -0.4 | 0.75 | negtive |
| Limonene | Pediococcus | -0.4 | 0.75 | negtive |
| Caryophyllene | Pediococcus | -0.4 | 0.75 | negtive |
| o-Cymene | Pediococcus | -0.4 | 0.75 | negtive |
| L* | Pediococcus | 0.4 | 0.75 | positive |
| Pediococcus | pH | 0.4 | 0.75 | positive |
| Hardness | Pediococcus | 0.4 | 0.75 | positive |
| Chewiness | Pediococcus | 0.4 | 0.75 | positive |
| Pentanal | Staphylococcus | 0.4 | 0.75 | positive |
| Heptanal | Staphylococcus | 0.4 | 0.75 | positive |
| Nonanal | Staphylococcus | 0.4 | 0.75 | positive |
| Octanoicacid | Staphylococcus | 0.4 | 0.75 | positive |
| Limonene | Staphylococcus | 0.4 | 0.75 | positive |
| Caryophyllene | Staphylococcus | 0.4 | 0.75 | positive |
| o-Cymene | Staphylococcus | 0.4 | 0.75 | positive |
| L* | Staphylococcus | -0.4 | 0.75 | negtive |
| pH | Staphylococcus | -0.4 | 0.75 | negtive |
| Hardness | Staphylococcus | -0.4 | 0.75 | negtive |
| Chewiness | Staphylococcus | -0.4 | 0.75 | negtive |
| 1-Pentanol | Pediococcus | 0.2 | 0.91666667 | positive |
| 2,3-Butanediol | Pediococcus | -0.2 | 0.91666667 | negtive |
| 1-Hexanol | Pediococcus | -0.2 | 0.91666667 | negtive |
| Hexanal | Pediococcus | 0.2 | 0.91666667 | positive |
| Benzaldehyde | Pediococcus | -0.2 | 0.91666667 | negtive |
| Aceticacid | Pediococcus | -0.2 | 0.91666667 | negtive |
| Butanoicacid,3-methyl- | Pediococcus | -0.2 | 0.91666667 | negtive |
| Acetoin | Pediococcus | -0.2 | 0.91666667 | negtive |
| Pediococcus | α-Pinene | 0.2 | 0.91666667 | positive |
| o-Xylene | Pediococcus | 0.2 | 0.91666667 | positive |
| p-Xylene | Pediococcus | 0.2 | 0.91666667 | positive |
| Anethole | Pediococcus | -0.2 | 0.91666667 | negtive |
| Gumminess | Pediococcus | 0.2 | 0.91666667 | positive |
| 1-Pentanol | Staphylococcus | -0.2 | 0.91666667 | negtive |
| 2,3-Butanediol | Staphylococcus | 0.2 | 0.91666667 | positive |
| 1-Hexanol | Staphylococcus | 0.2 | 0.91666667 | positive |
| Hexanal | Staphylococcus | -0.2 | 0.91666667 | negtive |
| Benzaldehyde | Staphylococcus | 0.2 | 0.91666667 | positive |
| Aceticacid | Staphylococcus | 0.2 | 0.91666667 | positive |
| Butanoicacid,3-methyl- | Staphylococcus | 0.2 | 0.91666667 | positive |
| Acetoin | Staphylococcus | 0.2 | 0.91666667 | positive |
| Staphylococcus | α-Pinene | -0.2 | 0.91666667 | negtive |
| o-Xylene | Staphylococcus | -0.2 | 0.91666667 | negtive |
| p-Xylene | Staphylococcus | -0.2 | 0.91666667 | negtive |
| Anethole | Staphylococcus | 0.2 | 0.91666667 | positive |
| Gumminess | Staphylococcus | -0.2 | 0.91666667 | negtive |
| aw | Pediococcus | 0 | 1 | aw |
| aw | Staphylococcus | 0 | 1 | aw |

# Supplementary Figure


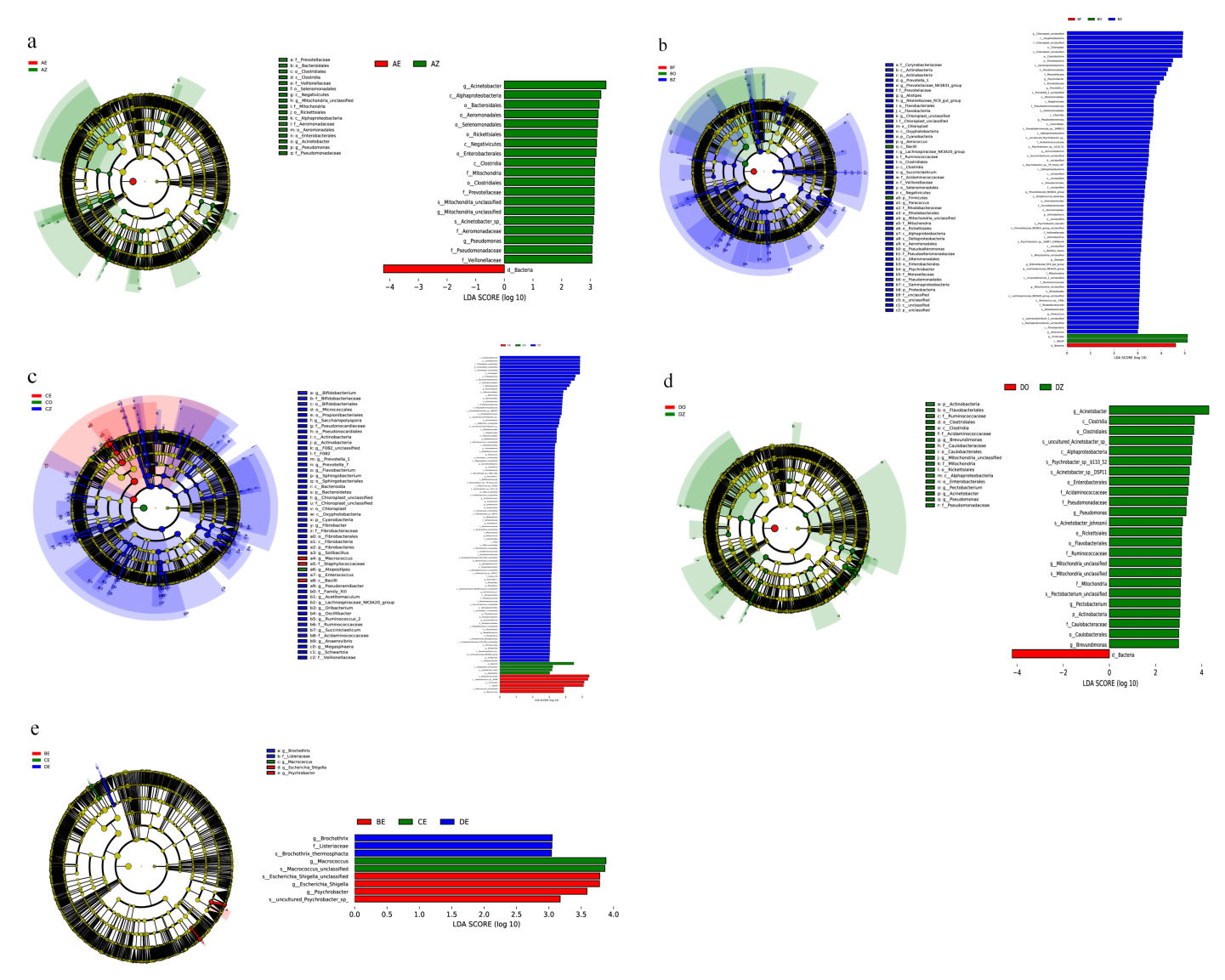


# Supplementary Figure 1 LEfSe species difference analysis and LDA score of samples. (A: CK group; B: M3 group; C: M5 group; D: M7 group; Z: fermented sausage was processed for 0 days in each group; O: fermented sausage was processed for 1 days in each group; F: fermented sausage was processed for 4 days in each group; E: fermented sausage was processed for 8 days in each group. Figure a, b, c, d: difference species between different groups in the same processing period. Only groups of statistically different species are shown in the figure.)
